# A Study on Maker Teaching Activity Design in Senior High School General Technology Course for Creativity Cultivation

**DOI:** 10.3389/fpsyg.2022.886051

**Published:** 2022-05-18

**Authors:** Hongjiang Wang, YuanFen Ye, Xiaoling Liao, Zuokun Li, Yingli Liang

**Affiliations:** ^1^School of Information Technology in Education, South China Normal University, Guangzhou, China; ^2^Tianhe Middle School, Guangzhou, China; ^3^School of Educational Science and Technology, Nanjing University of Posts and Telecommunications, Nanjing, China

**Keywords:** creativity, maker education, General Technology Course, senior high school, teaching model

## Abstract

General Technology Course (GTC) in senior high school focuses on skill training and the connection and comprehensive application of interdisciplinary knowledge, and it is a compulsory course for cultivating students' creative potential. However, GTC in domestic senior high school has low teaching efficiency and fails to cultivate students' creativity well. Fortunately, after years of theoretical and practical research in China, the Maker Education (ME), which focuses on cultivating students' innovative ability, has produced well-recognized applied research results. For this reason, this paper integrates the theories of ME into GTC. Combined the characteristics of ME and GTC, and followed the process of creation and the law of the expression of personality traits, we build a model of GTC based on ME to improve students' creativity effectively. In order to improve and optimize the designed teaching model, this study carried out three rounds of Action Research, designed the practical activities of GTC in senior high school, and revised the teaching model through action, observation and reflection continuously. Finally, this paper designed an experimental group and a control group. The experimental group adopts the recommended General Technology teaching model, and the control group adopts the traditional teaching model. Students were asked tested to take pre-test and post-test, and SPSS was used for analysis of ANCOVA and *T*-test. After analysis, the following experimental results were obtained: (1) the teaching model proposed in this paper can improve students' creativity significantly and effectively; (2) the adventurous, curiosity, imagination, challenge of students also have significant positive improvement.

## Introduction

In West, most educators agree that Technology Education should aim to help students develop an interest in technology and the ability to address technological challenges in a conscious and innovative way (Lind et al., 2020). For example, in Sweden, Technology Education is described as a discipline that aims to develop students' technical awareness and skills so that they can become part of and act in a technology-intensive world (SWEDISH National Agency for Education [Skolverket], 2017). However, Technology Education still suffers from problems such as teachers' differing views on what should be covered in curriculum topics (Norström, 2014) and the inability to achieve continuity between what policy documents require and the realities of practice (Doyle et al., 2019). In this case, the effect of cultivating students' creativity through Technology Education is not significant. The Technology Education in this study is the General Technology Course (GTC) currently implemented in senior high schools in China. It is a course whose goal is cultivating students' creativity through “learning by doing” and practical experience (Ministry of Education of the People's Republic of China, 2017). However, General Technology Course (GTC) teaching in China also has some problems, such as single teaching form, backward teaching content and equipment, and insufficient teaching efficiency in course, which led the course fail to cultivate students' creativity (Gu, 2014).

Creativity is considered one of the four century skills of twentyfirst century. The researches on creativity have particularly flourished in recent decades (Nenad and Limin, 2017). Research shows that using maker education may be very suitable for classroom learning (Kim and Kim, 2018). Frank (1971) believes that the creative individual has the psychological characteristics of curiosity, adventurous, challenge and imagination. Surprisingly, the improvement of creativity is not a prominent issue in schools. In order to solve the problem that the General Technology Course fails to effectively cultivate students' creativity, some scholars have conducted research on this. Lv (2016) respectively proposed methods to cultivate students' creativity in GTC, such as exploring products in life, creating democratic classrooms to stimulate students' imagination, and using design activity carriers. Xiong (2016) proposed to cultivate students' innovative ability through the diversification of design projects, such as students' independent choice of project themes and reasonable arrangement of project design task time.

Noted that few scholars have carried out research on GTC teaching model for the cultivation of students' creativity. In recent years, the theory and practice of Maker Education (ME) have developed rapidly, and some excellent applied research results have emerged. The core educational value of ME is student-centered, with project practice as the carrier to cultivate students' innovative ability (Yang and Li, 2015). ME is seen as a way to enhance future capabilities (Seo and Lee, 2018). Students engage in production activities and develop a maker mindset through ME (Martin, 2015). The maker mindset is seen to be related to important competencies such as innovative ideas and actions (Kang, 2017), critical thinking, creativity, problem solving and collaboration (Kang and Yoon, 2017), etc. ME has the advantages of openness, compatibility, sharing, and practicality, which helps to cultivate students' creative ability (Yang et al., 2019). In addition, ME is closely related to the learning principle of constructivism, which also emphasizes “learning by doing” in educational theory (Kim, 2018; Yoon, 2018; Kim et al., 2020). Moreover, ME focuses on the learning process, encourages students to divide labor according to their personal interests and expertise, and turns creativity into reality through hands-on practice. Therefore, the fusion of GTC and ME to better cultivate students' creativity has great possibilities for teaching practice. However, few scholars have carried out research on this at present. You (2017) designed a maker teaching case called the Arduino robot making in the GTC, which improved students' technical literacy. The researches about teaching model, teaching activity cases, and teaching effect on the integration of GTC and ME to cultivate creativity is relatively rare. Therefore, this paper designs a GTC teaching model based osthe concept of ME for creativity cultivation. Under the guidance of the model, we design teaching activities and carry out experimental research to cultivate students' creativity effectively.

The research purposes of this study are: (1) to construct a GTC teaching model based on the concept of ME. (2) to apply this model to practice and evaluate whether it can effectively improve students' creativity.

## Construction of GTC Teaching Model Based on the Concept of ME

### Four Periods of Creative Process

The creative process proposed by Wallas (1926) should go through four stages: preparation, gestation, enlightenment and verification. The thinking operation in the preparation period is cognitive memory, and the personality traits are studious, diligent, and maintaining attention. the creator in gestation period is bold and imaginative. In enlightenment period, the creator is enlightened by epiphany or by discovering a solution to the problem. The personality traits manifest as taking risks and accepting failure in this period. In verification period, the creation plan will be verified by facts. Therefore, according to the four periods proposed by Graham Wallas, this study divides the creative process of students in General Technology curriculum into four periods: preparation, gestation, enlightenment and verification.

### Five Stages of Creative Problem Solving

Creative problem solving (CPS) model is a learning and teaching model that can effectively cultivate students' creativity. Research on creativity development shows that the most effective way to cultivate creativity is to use the CPS model (Torrance, 1972). Therefore, this study intends to introduce CPS model into the design of teaching model. Parnes (1967) proposed the five stages of CPS firstly, which divided the problem solving process into five steps: discovering facts, discovering problems, seeking ideas, seeking solutions and seeking acceptance. Stanish and Berle (1997) proposed that the creative problem solving process includes six procedures: finding confusion, collecting data, finding problems, collecting ideas, looking for countermeasures and accepting ideas. Therefore, referring to the above process, this study integrates the concept of ME into the GTC and designs the teaching process with CPS model, including seven links of “finding problems, condition evaluation, clarifying problems, formulating schemes, design and production, communication and sharing, evaluation and reflection.”

### Maker Education Activities

Fu (2015) proposed the “SCS Maker Teaching Method,” which divides the teaching into seven steps, introduction of sentimental stories, imitation of simple task, explanation of key points of knowledge, imitation of extended task, stimulation and guidance of innovation, collaborative task completion, and sharing of successful works. Zhu and Hu (2016) designed a design-based learning model for Maker Education, in which teachers' activities include determining projects around themes, presenting scenario and describing challenges, establishing standards and providing supports, supervising and observing timely guidance, organizing cross-border cooperation in learning, publishing results, evaluation and reflection. At present, there is no unified model for ME, but from the common characteristics of the above-mentioned model, it can be seen that ME should be based on a certain situation, let students experience learning and “learning by doing,” and share the joy of the work finally. Therefore, this study refers to the maker teaching steps proposed by the above scholars, and sets the teacher's activities as “case display to stimulate thinking, guide students to evaluate projects, formulate evaluation standards objectively, guide students to design schemes, guide students to practice, organize students to exchange and share, carry out diversified assessments.” According to the teacher's activities, the students' activities are set as “contact life and think actively, understand the feasibility and scientificity of the project, group discussion and evaluate project objectively, cooperate to draw design sketches, practice and solve problems, report and exchange, inter-group and intra-group evaluation.”

**Figure 1:**
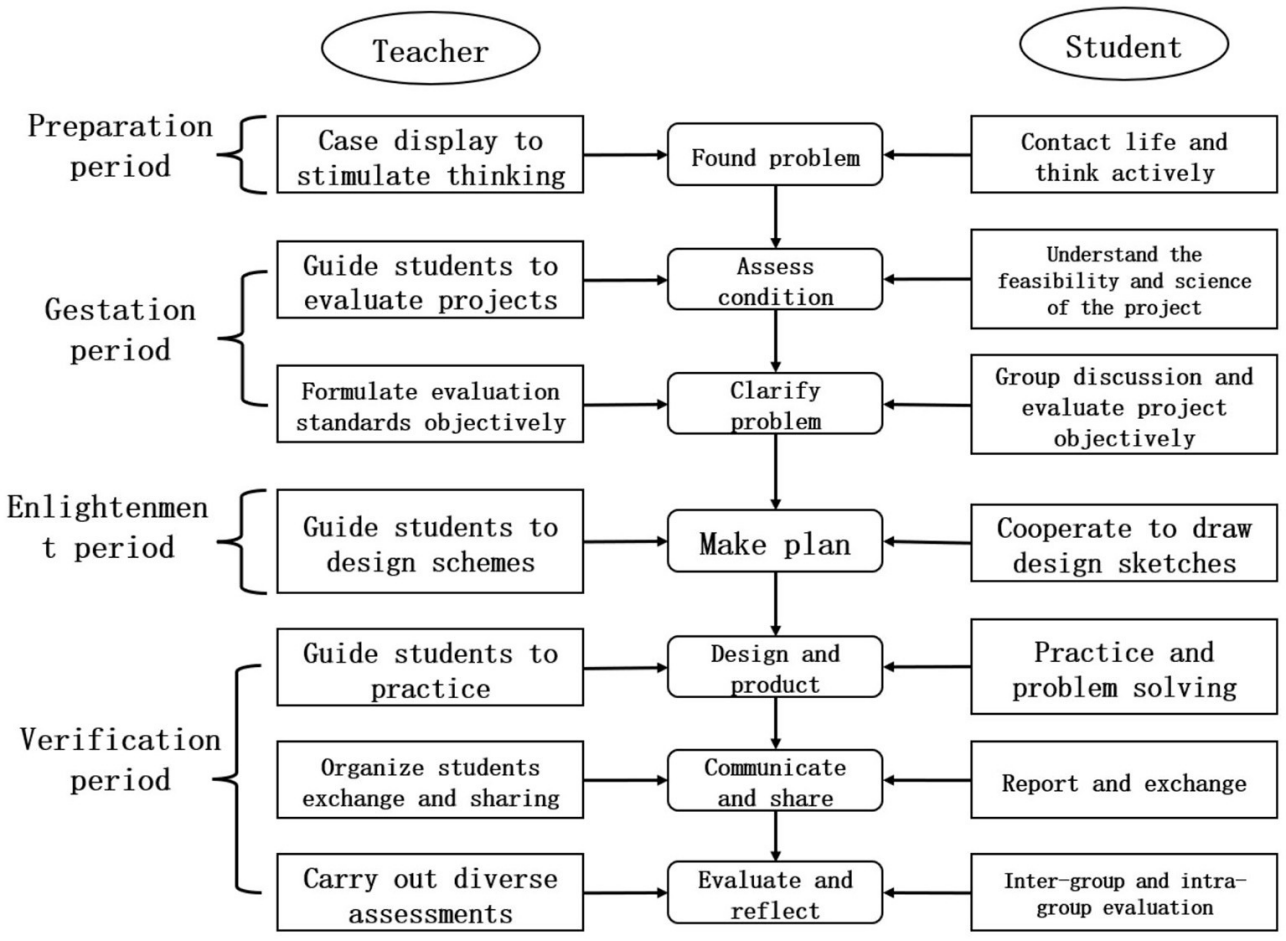
General Technology Course teaching model based on the concept of maker education (original).

Based on the above analysis, the paper constructs a GTC teaching model based on the concept of ME as shown in Figure 1. This teaching model focuses on combining with students' actual life. Firstly, students discover problems in life observation. Secondly, they evaluate objective and actual conditions reasonably, and then establish the project theme, formulate a feasible design plan through group discussion, and then carry out design and production. In the process, students think actively, solve problems and communicate after completing the works. Finally, students evaluate the works of this group and other groups objectively.

## Action Research on GTC Based on ME Concept

This research adopts Action Research (AR). AR is a reflective inquiry activity carried out by participants in social situations, which combines “action” and “research” in order to improve practice and rationally understand practical activities and their environment (Kemmis and Mctaggart, 1982; Carr and Kemmis, 1986). AR advocates “teachers as researchers” to find and solve problems in the real educational environment. It opposes separating phenomena from situations and attaches importance to “learning by doing” in real situations. In AR, through the circular chain of “plan, action, observation, reflection,” the researchers improve action constantly, so as to deepen the research and achieve the purpose of improving practice. The reason why this study adopts AR rather than experimental research in traditional education research is: in order to implement and develop the new teaching model and method, it must be rooted in the real soil, rely on the test of teaching practice in the real situation. The paper adopt AR, and constantly find, analyze and solve problems in the real teaching environment, so as to continuously improve the teaching mode proposed in this study. In addition, the openness and dynamics of the spiral cycle process of AR “plan, action, observation, reflection” is in line with the process of repeated exploration in the design of teaching mode and the implementation of teaching scheme.

Therefore, this study selected 43 senior one students from a high school with a history of teaching general technology for many years in Guangzhou as the object of AR, and designs three rounds of General Technology Course (GTC) practice activities based on the concept of Maker Education (ME). Each round of AR aims to foster student creativity. The teaching content focuses on the integration of ME and GTC, and includes three projects, i.e., “The Production of Lamp Painting,” “The Production of Sound and Light Alarm System” and “The Production of Laserblock-based Arduino.”

The teaching practice is scheduled for the first semester of the academic year 2021-2022. The specific period is from September 6 to December 31, 2021. The total number of hours is 16. The first round of AR was conducted from September 6 to September 30, 2021, for four class periods, one 45-mins class period per week. The first round of AR adopted the original teaching model shown in Figure 1, and conducted the first round project “The Production of Lamp Painting,” which corresponds to the content of the compulsory 1 “General Process of Technical Design” chapter in GTC, to examine and analyze the effectiveness of the teaching model in fostering students' creativity, as well as what problems exist. The second round of AR was conducted from October 11 to November 12, 2021, and consisted of five class periods. The instructional content was the second round project “The Production of Sound and Light Alarm System,” which corresponded to the content of the compulsory 2 “Process and Design” and “System and Design” chapters. The goal of this round of AR was to put the second round of the generic technology-based Maker teaching model into new AR practice, to observe and analyze the effectiveness of the improved second round teaching model in this round of teaching AR, and what problems existed. The third round of AR was held from November 15 to December 31, 2021, and consisted of seven class periods. The teaching content was the third round project “The Production of Laserblock-based Arduino,” which corresponded to the compulsory 2 “Structure and Design” chapter. The modified generic technology-based creator teaching model after the problems identified in the second round of AR practice was put into the third round of AR practice to observe and analyze the pedagogical effects of the improved third round model obtained in this round of teaching AR.

In each round of AR, it is necessary to obtain information such as students' learning attitude, enthusiasm and completion of classroom tasks through classroom observation, next reflect on the existing problems of the current round of teaching model, then put forward improvement measures to optimize this round of teaching mode, and finally put the improved new teaching mode into the next round of AR. Due to the space limitation of the article, this paper only gives the detailed teaching activity design of the third round of AR (as shown in Table 1).

**Table 1:** The teaching activities of the third round of action research.

**Teaching process**		**Teacher activity**	**Student activity**	**Purpose of design**
Preparation period	Found problem	The videos of “Laserblock-based Arduino Production” made by previous students are selected for display to introduce the learning content and goals of this project. Question: According to the Arduino function modules you have learned, what kind of comprehensive project with structure do you want to design?	Students watch the video and think based on learning objectives and content proposed by the teacher.	To link the content learned and mobilize the divergent thinking of students.
Gestation period	Assess condition	The teacher asks the evaluation points of relevant projects made by previous students, and analyze the scientificity and feasibility of the design themes proposed by students.	Students think about the main points of project evaluation, and evaluate design problems reasonably and objectively in combination with evaluation criteria.	To improve students' enthusiasm for active thinking, and enhance students' recognition of the evaluation standards formulated later.
	Clarify problem	The teacher guides students to jointly formulate project evaluation standards and comprehensively evaluate design issues.	Through group discussions, students can objectively judge the project conditions and determine the design theme according to the evaluation criteria.	To enhance students' awareness of the feasibility of designing problems.
	Knowledge and skills learning	The teacher designs a technical experiment that affect structural stability and strength factors, and guides students to think about the basic ideas and methods of structural design.	Technology test exploration.	Let students learn the basic knowledge module to lay the foundation for the subsequent program design and production.
Enlighten period	Make plan	The teacher and students formulate program forms jointly and program evaluation standards, and use the Zhixin System to feedback program evaluation results.	Students draw the project design flow chart and try to summarize the elements of the design scheme and the key points of the scheme evaluation.	To enhance students' learning subject awareness.
Verification period	Design and product	The teacher give timely guidance to students with learning difficulties, and issue the previous knowledge and skills micro-courses and common problem-handling micro-courses.	Students carry out practice according to the division of labor in the plan, actively think about problems encountered in the process of practice, and find ways to solve problems.	To cultivate students' creativity and hands-on ability
	Communicate and share	The teacher and students work together to formulate evaluation criteria for works. Teacher organizes students to demonstrate project works.	Students try to summarize the evaluation dimensions of the works, and the group takes the stage to display the lamp painting works.	To enhance students' recognition of evaluation criteria
	Evaluate and reflect	The teacher organizes students to evaluate their works in groups, and guides students to reflect on the evaluation criteria.	Students objectively evaluate the work of this group and other group works according to the evaluation criteria	To enable students to improve their creativity through self-reflection

After three rounds of AR, after reflecting on the existing problems, the following modifications were made to the teaching model: (1) In “Discover Problem,” a new system was introduced. “Zhixin Online Teaching Evaluation System” (Zhixin System) is developed by the team of a teacher in Guangzhou No.6 Middle School for teaching management and evaluation. The introduction of Zhixin System hopes to correct the learning behaviors of students in the first round of AR, such as not bringing books to class but bringing snacks and drinks. (2) In “Explicit Conditions,” the case of previous student projects is introduced to guide students to evaluate projects. Showing actual cases close to students' learning tasks is conducive to further stimulating their curiosity in learning and improving the feasibility of students' projects. (3) In “Identify Questions,” teachers will show the project evaluation standards, and then teachers and students formulate the standards for this project activity jointly. Allowing students to achieve scientific evaluation according to evaluation criteria is good to improve the feasibility of the project. (4) A new activity “Knowledge and Skills Learning” is added to allow students to learn the relevant knowledge and skills, such as open source software and hardware. When formulating a design plan, they can specifically describe the functions of the works in the plan. It is also helpful to make a clearly task division in the group. In addition, teachers have added the activity of “making micro-lectures” to make the content of knowledge and skills involved in the project into micro-lectures for students to learn. (5) In “Make Plan,” group work is added. Students divide the project tasks reasonably according to the respective strengths of the members of the group, so that everyone in the group has something to do and enhance the cohesion of the group. Introduce thinking tools to assist students in drawing design sketches. In addition, increase student self-assessment, so that students can learn self-assessment and reflection. (6) In “Design and Product,” the timely feedback of Zhixin System is introduced. Using the “class record” function of Zhixin System, teachers can add or subtract points and give feedback to students in real time, which may improve students' enthusiasm for learning. (7) In “Communicate and Share,” students participate in the formulation of work evaluation standards to enhance recognition of the standards and participation in the classroom. (8) In “Evaluate and Reflect,” use Zhixin System to add students' personal evaluation. Students can be bold to make objective evaluations of others' works, which may cultivate their sense of adventure. At the same time, teacher can quickly collect students' evaluation opinions, and calculate the evaluation scores of each group timely.

**Figure 2:**
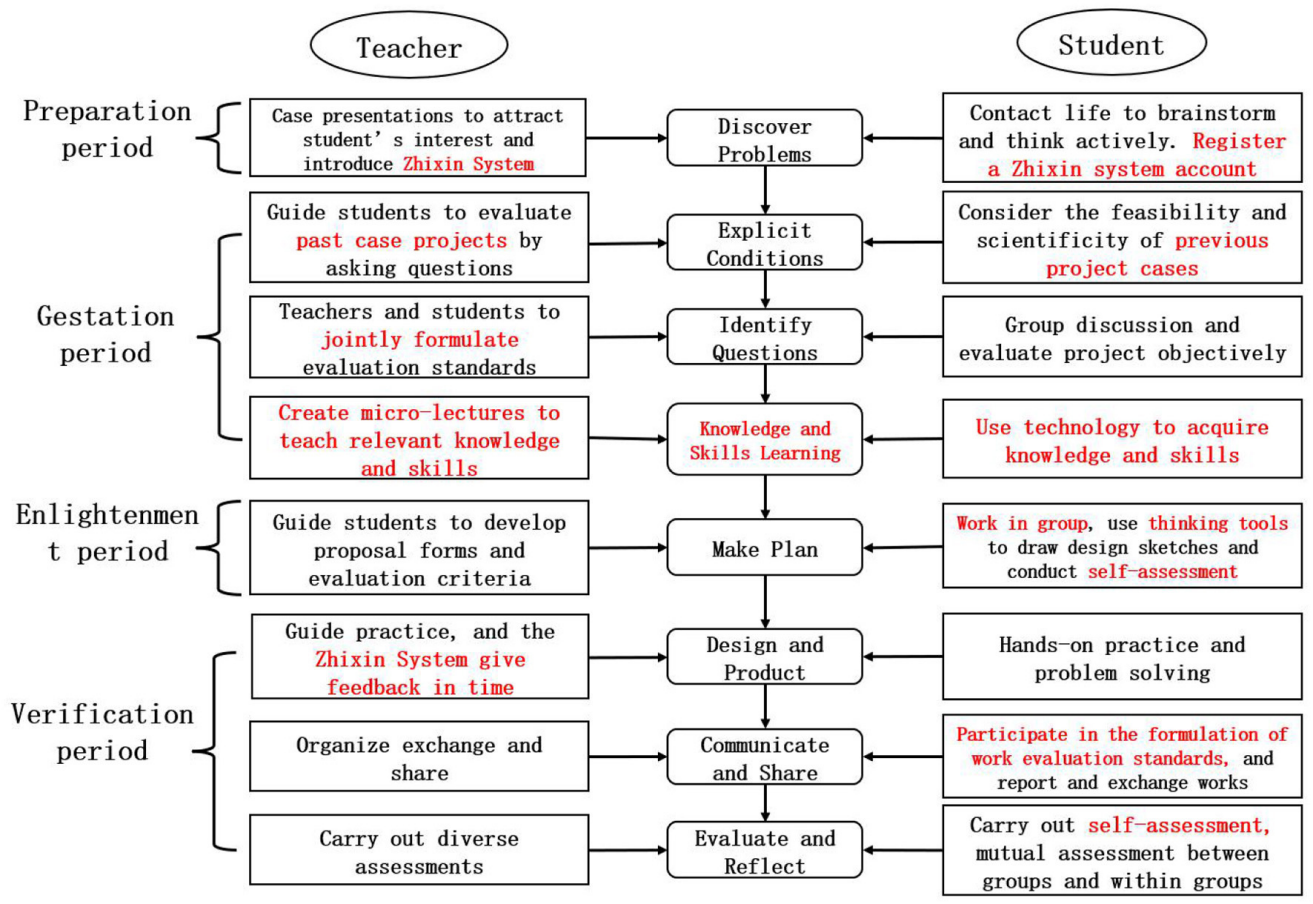
General technology course teaching model based on the concept of maker education (after action research).

It can been seen that after three rounds of AR, the original teaching model introduced new technologies (such as open-source software and hardware), new systems (such as Zhixin Online Teaching Evaluation System, referred to as “Zhixin System”), and added new teaching activities (such as, knowledge and skills learning). We observed and found that students' performance in the class and learning performance were relatively positive. At the same time, through modification and improvement, a relatively complete GTC teaching model based on the concept of ME has been obtained (as shown in Figure 2).

The teaching model divides the teaching process into seven steps: Discover Problems, Explicit Conditions, Identify Questions, Knowledge and Skills Learning, Make Plan, Design and Product, Communicate and Share and Evaluate and Reflect. Combining the four periods of preparation, gestation, enlightenment and verification proposed by Wallas (1926) in the creation process, each step is divided into a specific period, and different teaching activities are carried out according to the characteristics of students and the concept of Maker Education. In “iscover Problems,” the teacher stimulates students' brainstorming and active thinking through case presentations, and introduces new systems to assist teaching. In “Explicit Conditions,” teacher shows case projects from previous years, and guides students to think about the feasibility and scientificity of those projects. In “Identify Questions,” students make objective group evaluations of previous projects through group discussions. On this basis, the teacher and students jointly develop project evaluation standards. In “Knowledge and Skills Learning,” the teacher prepares relevant micro-lectures before class for students to learn. In addition, teacher should prepare more forms of teaching materials, and educate students to learn to solve problems and learn knowledge by themselves. In “Make Plan,” teachers guides students to carry out activities and conduct self-assessment. Through group cooperation, students are good at drawing design sketches by using mind map tools. In “Design and Product,” students practice according to the developed plan. The teacher is informed of the students' situation in a timely manner and give feedback on Zhixin System. In “Communicate and Share,” teacher and students jointly formulate the evaluation criteria for works, and students report and exchange their works. In “Evaluate and Reflect,” teacher organizes multiple evaluations, such as mutual evaluation within the group, mutual evaluation between groups, individual self-evaluation, and teacher evaluation.

## Analysis of the Application Effect of GTC Teaching Model Based on the Concept of ME

### Experimental Procedure

The GTC teaching model based on the concept of ME has been improved through AR. In order to verify the effect of this teaching model on improvement of students' creativity, this study adopts the quasi experimental research method. In this study, some freshmen in a senior high school in Guangzhou, China was selected to set up an experimental group (23 males and 20 females) and a control group (20 males and 17 females), and then we carry out a five-month teaching practice process to explore the effect of the recommended teaching mode. The experimental hypotheses designed in the paper are that: (1) the teaching activities designed under the guidance of the general technology-based creative teaching model can effectively improve students' creativity; (2) the teaching of general technology under the traditional teaching model is not effective in enhancing students' creativity. The adopted independent variable is the teaching activities guided by the general-purpose technology-based creator teaching model. The dependent variable used is students' creativity (based on the Williams Creativity Tendency Test). The control variables used are the same level of creativity of the students in the experimental class and the control class before the experiment, and the same teacher in both the experimental and control classes.

A pre-test was carried out before the experiment, and no significant difference was found between the two groups *(Sig* = *0.064*>*0.05)*. When the experiment was carried out, the experimental group adopted the GTC teaching model based on the concept of ME, and the control group adopted the traditional teaching model. After the experiment, the students in the experimental group and the control group were get a post-test of creativity. Finally, the collected data are analyzed by SPSS.

Regarding the test of students' creativity level, the scale used in this study is the well-known Williams Creativity Tendency Test, i.e., the *Williams Prefer Measurement* (WPM) *Forms* (Williams, 1993). The scale, which has good reliability and validity, was originally developed by Williams. In 1999, Taiwanese scholars Lin and Wang (1999) re-tested the reliability and validity of the scale in primary and secondary schools in Taiwan, and the re-test results showed that the scale had good reliability and validity. It has been widely used by researchers in various industries as a creativity test scale in various learning contents for primary, secondary and university students. Therefore, in order to demonstrate fairness and objectivity, we used the accepted WPM forms instead of other methods to verify the validity of our findings. The WPM forms includes all four dimensions that we are measuring. The scale consists of 50 questions, each of which can be selected from three options: “completely agree” (3 points), “partially agree” (2 points), and “completely disagree” (1 point). The scale includes four dimensions: adventurous, curiosity, imagination, and challenge. After the weighted calculation, the total score of the individual test can be obtained. The higher the individual's total score, the higher the level of creativity. After test, the coefficient of internal consistency and validity of structure for the perception survey of all participating students was acceptable with Cronbach's alpha equal to 0.915, KMO equal to 0.950. Therefore, we conclude that the perception survey was valid and reliable.

### Data Analysis

The data analysis process includes: in order to verify the teaching model proposed in the research, firstly, data were analyzed by using analysis of covariance (ANCOVA) to determine any significant differences between the experimental and control groups by post-test scores with the previous test as a covariate. Sencondly, data were analyzed by using Paired sample *T*-test to determine any significant differences between pre-test and post-test in experimental group or control group. The assumptions of ANCOVA were first checked to ensure that they were met in the analysis of covariance for these studies. Tests of the assumptions for ANCOVA and inferential statistical analyses were carried out using SPSS (Statistical Package for Social Sciences version 26.0). The results show no interaction between covariates and independent variables *(F* = *2.621, p* = *0.110* > *0.05)*.

#### ANCOVA Between Experimental and Control Groups

Table 2 shows the results of ANCOVA and descriptive data analysis on students' creativity post-test scores. It is statistically indicated that the course taught using the GTC Teaching Model Based on the Concept of ME scored significantly higher than those taught using the traditional teaching method *(F* = *405.301, p* <* 0.00)* (total items). Additionally, significantly higher achievement scores for the experimental group were also found at the Adventurous *(F* = *171.981, p* <* 0.00)*, Curiosity *(F* = *141.191, p* <* 0.00)*, Imagination *(F* = *113.165, p* <* 0.00)*, and Challenge *(F* = *116.885, p* <* 0.00)*. This shows that the GTC teaching model based on the concept of ME has significantly improved the creativity of students, including four dimensions of adventure, curiosity, imagination, and challenge.

**Table 2:** Summary of ANCOVA on students' creativity.

**Post-test level**	**Experimental group (SD)**	**Control group (SD)**	**F**
Adventurous	27.446 (0.106)	25.401 (0.114)	171.981^**^
Curiosity	36.029 (0.156)	33.290 (0.168)	141.191^**^
Imagination	30.691 (0.164)	28.089 (0.177)	113.165^**^
Challenge	31.072 (0.155)	28.565 (0.168)	116.885^**^
Total items	125.254 (0.331)	115.327 (0.358)	405.301^**^

** *p < 0.01*.

#### Paired Sample *T*-Test Before and After Experiment

Based on SPSS (version 26), a paired sample *T*-test was performed on the pre-test and post-test of the experimental group and control group. The values obtained after the analysis are shown in Table 3. The results showed that there was an extremely significant difference between the pre-test and post-test of creativity in the experimental group. There was a significant difference in the control group. In the four dimensions of adventure, curiosity, imagination, and challenge, the experimental group also had extremely significant differences before and after the test, while the control group only had significant differences in imagination. This proves that traditional GTC can improve students' creativity, focusing on the improvement of imagination. By incorporating ME concepts and activities into GTC, students' creativity has been greatly improved. And the four dimensions of students' sense of adventure, curiosity, imagination and challenge have also been extremely significantly improved. This shows that the GTC teaching model based on the concept of ME is more conducive to the cultivation of students' creativity.

**Table 3:** Paired sample *T*-test for each dimension before and after the test.

		***N***	**Mean**	**Sig**.
Experimental group	Creativity in pre-test	43	112.16	0.000^**^
	Creativity in post-test	43	124.91	
	Adventurous in pre-test	43	25.00	0.000^**^
	Adventurous in post-test	43	27.49	
	Curiosity in pre-test	43	32.56	0.000^**^
	Curiosity in post-test	43	36.35	
	Imagination in pre-test	43	26.65	0.000^**^
	Imagination in post-test	43	30.30	
	Challenge in pre-test	43	27.95	0.000^**^
	Challenge in post-test	43	30.77	
Control group	Creativity in pre-test	37	117.59	0.011^*^
	Creativity in post-test	37	118.05	
	Adventurous in pre-test	37	25.70	0.160
	Adventurous in post-test	37	25.76	
	Curiosity in pre-test	37	33.73	0.096
	Curiosity in post-test	37	33.86	
	Imagination in pre-test	37	29.00	0.044^*^
	Imagination in post-test	37	29.11	
	Challenge in pre-test	37	29.16	0.083
	Challenge in post-test	37	29.32	

* *p < 0.05*;

** *p < 0.01*.

## Conclusions and Discussions

This research firstly integrates the theory of ME into GTC, and constructs the GTC teaching model based on the concept of ME. Secondly, through three rounds of AR, the teaching model is optimized. Finally, the quasi-experimental research method was used to apply the teaching model to teaching, which verified the feasibility and effectiveness of it. It found that the teaching model has greatly improved the creativity of students, and increased their sense of adventure, curiosity, imagination, and challenge greatly. The main reasons are as follows:

(1) Adventure: The implementation of the GTC teaching model based on the concept of ME has created a “learner-centered” learning environment. The ME practice project encourages teachers to implement problem-based teaching, requires students to break the passive learning model under the traditional teaching system, and cultivates the ability to accept new knowledge and explore (Kai-Han et al., 2019).
(2) Curiosity: The implementation of the ME model has brought new technologies and new systems to students, such as open-source software and hardware and Zhixin System, as well as knowledge and technologies outside the major, driving students' curiosity (Kai-Han et al., 2019).
(3) Imagination: We have developed some projects to hone students' innovative thinking, such as brainstorming. In the third round ofAR, thinking tools are introduced for design sketching, such as “Mind Map” and “Concept Map.” These are all conducive to the divergent and convergent thinking of students and the main part of imagination cultivation (Moorman et al., 2017).
(4) Challenge: The GTC in this study integrates multi-disciplinary content, including open-source software and hardware knowledge, programming knowledge and mathematics knowledge. New knowledge, new technologies, and new teaching model may impose cognitive load on students (Shadiev et al., 2019), thereby creating challenges for students. In general, under the support of the ME concept, students go through preparation, gestation, enlightenment, and the verification period in the teaching, and continue to design, produce, share, and communicate with their works, which improves their creativity. Some scholars have pointed out that creating meaningful products or social outcomes is the most effective learning experience (Kang and Yoon, 2017; Yoon, 2018). ME structures and systems knowledge and experiences as learners generate deliverables and share experiences (Kim et al., 2022). Thus, it is the integration of ME into GTC to create valuable products and share them through exchanges, which will help students master knowledge and improve creativity better.

## Applications and Perspectives

Practical application value of this research:

(1) Through three rounds of teaching AR, the Maker teaching mode based on the general technology can be used in General Technology curriculum teaching practice of cultivating students' creativity.
(2) Maker teaching model based on general technology built in the paper can effectively improve students' creativity, cultivate the students' creative potential. The experimental results show that experimental class students not only master the basic knowledge and skills of general technology, master the general process and method of technical design, but will be trying to find a way to solve problems on their own when they encounter problems, which means that integrating the concept of ME into the General Technology curriculum has a great effect on the cultivation of creativity including adventure, curiosity, imagination and challenge.
(3) The proposed Maker teaching model has certain application value to promote cultivating students' creativity in General Technology curriculum in the practice of teaching activities. Teachers should pay attention to the implementation of all aspects of GTC in daily teaching to enhance creativity.

However, due to factors such as time, space and resources, this study still has some shortcomings. Future research could focus on the following points:

(1) This study did not analyze the effect of the recommended creative teaching model for developing learners' creativity on the gender of male and female students. There is some variability in the creativity of boys and girls. It is necessary for a deeper study on the impact of boys and girls, which is a direction for future research.
(2) The general technology-based creative practice activities are relatively single. Due to the heavy academic burden of high school students and the limited time for extracurricular learning, the authors only designed the Arduino creator practice activities based on universal technology in this study. Because of the single practice activity, the persuasive power that the universal technology-based Maker teaching model can effectively enhance students' creativity is not enough. In the next study, other generic technology-based Maker teaching activities are added, and the generic technology-based maker teaching model is further improved in more practical activities.
(3) Creativity results were only tested using a single scale and lacked a complete test of all aspects of creativity in the paper. In the follow-up research, theoretical tests and practical operation tests should be added to the test of students' creativity. The creativity test scale should be improved to reflect the creative personality traits of adventure, curiosity, imagination and challenge on the basis of the Williams Creativity Tendency Test for the specific content of practical activities, so that the creativity test scale can be more relevant and convincing. Furthermore, enriching creativity testing methods and diversifying creativity evaluation methods make the research results more reasonable.

## Data Availability Statement

The original contributions presented in the study are included in the article/supplementary material, further inquiries can be directed to the corresponding author/s.

## Ethics Statement

Ethical review and approval was not required for the study on human participants in accordance with the local legislation and institutional requirements. Written informed consent from the patients/participants was not required to participate in this study in accordance with the national legislation and the institutional requirements.

## Author Contributions

HW was responsible for thesis architecture design, theoretical design, implementation plan design, and thesis writing. YY was responsible for the implementation of three rounds of action research teaching activities and data collection. XL was responsible for classroom design and classroom observation and recording. ZL was responsible for diagram design and data processing, as well as thesis revision. YL was responsible for the investigation of relevant research status at home and abroad, including the research status of general technology teaching, maker education, and creativity cultivation. All authors contributed to the article and approved the submitted version.

## Funding

This study was financially supported by Research Funds of 2017 Youth Foundation Project for Humanities and Social Sciences Research of the Ministry of Education (17YJC880098).

## Conflict of Interest

The authors declare that the research was conducted in the absence of any commercial or financial relationships that could be construed as a potential conflict of interest. The handling editor declared a shared affiliation, though no other collaboration, with several of the authors, HW, XL, and ZL, at the time of the review.

## Publisher's Note

All claims expressed in this article are solely those of the authors and do not necessarily represent those of their affiliated organizations, or those of the publisher, the editors and the reviewers. Any product that may be evaluated in this article, or claim that may be made by its manufacturer, is not guaranteed or endorsed by the publisher.
